# Seasonal and Weather Effects on Rheumatoid Arthritis: Myth or Reality?

**DOI:** 10.1155/2020/5763080

**Published:** 2020-09-07

**Authors:** Hamida Azzouzi, Linda Ichchou

**Affiliations:** Rheumatology Department, Mohammed VI University Hospital, Faculty de Medicine, Mohammed I University, Oriental, Oujda, Morocco

## Abstract

**Introduction:**

Many of our rheumatic patients report that weather and seasons affect their symptoms.

**Objective:**

The purpose of this study was to examine the effect of meteorological parameters within seasons on rheumatoid arthritis (RA) symptoms.

**Methods:**

A retrospective longitudinal study from July 2017 to August 2018 was conducted. Data from three consultations for three seasons were collected and included a tender and swollen joint count, a disease activity score for 28 joints (DAS28), and patient's pain assessment from their computerized medical record. The weather conditions (minimum and maximum temperature, precipitation, humidity, atmospheric pressure, and wind speed) registered during the same day of consultation for each patient were obtained. Then, the statistical correlation between each meteorological parameter and RA parameters was determined using the multiple linear regression analysis.

**Results:**

The data of 117 patients with a mean age of 50.45 ± 12.17 years were analyzed. The mean DAS28 at baseline was 2.44 ± 0.95. The winter in Oujda is cold (average temperature between 10°C and15°C) compared to summer (24.5°C–32.7°C). The spring is wetter with a 71% average humidity. Overall, the tender joint count was significantly correlated with hygrometry (*p*=0.027) in winter. A similar result was obtained in summer with precipitation (*p*=0.003). The pain intensity in the summer was negatively correlated with minimum temperatures and atmospheric pressure. However, there was no correlation between meteorological parameters and disease objective parameters for all seasons. Multiple linear regression analysis showed that weather parameters appeared to explain the variability in four RA predictors in the summer. No significant associations were observed in the spring.

**Conclusion:**

Our study supported the physicians' assumption regarding the effect of climate on pain in RA patients.

## 1. Introduction

The effect of climate on rheumatoid arthritis (RA) symptoms is frequently addressed by our patients. There are conflicting results in the literature, and it is unclear whether the effect is a coincidence or a direct effect. Among the arguments advanced to explain the inconclusive findings reported on the climate effect on pain, we find seasons. Seasons are defined by climate at a particular place on the earth. Seasonality is a phenomenon that has been frequently reported to be related to various conditions in humans, including diseases [1, 2], growth [3], gene expression and immunity [4], and physiology [5, 6]. Specifically, the effect of seasonality on autoimmune and joint diseases has been described in the studies [7, 8]. For rheumatoid arthritis, this effect has been described for the onset of the disease, its course, and even its radiographic progression and severity [9].

Furthermore, symptom and particularly pain analysis is one of the difficulties encountered among the studies on weather effects on arthritic patients. Indeed, pain is difficult to objectify as it is a subjective experience with considerable inter- and intraindividual variations. Moreover, even if the study on weather parameters is regarded to be simple, the interrelation between weather parameters and human beings is complex; in addition, seasonal variations should be taken into consideration [10].

Fortunately, in recent years, the identification and evaluation of RA have become more accessible and more precise. Furthermore, during the last decade, the treatment of RA has undergone considerable evolution; thus, our patients are usually in remission. Few studies have addressed this question after the era of disease activity score (DAS28) within the current state of the art.

The specific peculiarities of climate from different geographic areas could also be a possible explanation for controversies found within reports. The climate of Oujda city—located to the northeast of Morocco—is classified as dry (steppe climate) with an average temperature of 16.7°C and an average annual precipitation of 338 mm [11] (http://www.fr.climate-data.org).

Thus, we decided to evaluate the seasonal effect of meteorological parameters on our RA patients' symptoms.

## 2. Patients and Methods

### 2.1. Study Design

We performed a retrospective longitudinal study on our RA patients at the rheumatology department of Mohammed VI University Hospital at Oujda city. The study period was between July 2017 and August 2018. The data from three consultations, each of which corresponded to a defined season (i.e., winter, spring, and summer) for every patient, were obtained from patients' electronic medical records. The meteorological parameters during the day of every clinical evaluation were recorded. The requirement of informed consent was waived owing to the retrospective nature of the study.

### 2.2. Subjects

The patients were included if they were diagnosed with rheumatoid arthritis according to the American College of Rheumatology and the European League Against Rheumatism (ACR-EULAR) criteria of RA (2010) and if their data were available for three seasons. These patients were receiving RA treatments according to national recommendations (mainly methotrexate, steroids, and rituximab as the first biological drug). Patients with a very active disease (DAS28 > 5.1), which may have needed RA therapy adjustment and those with unavailable data on more than two seasons, were excluded.

### 2.3. Meteorological Parameters

Data on the meteorological conditions during the same day of consultation for each patient were obtained from the ANGADs Airport Weather Center (i.e., the nearest station at the altitude of 470 m). Minimum and maximum temperatures were assessed in degrees Celsius (°C). Precipitation on the day of the visit was indicated in millimeters (mm), atmospheric pressure in millibars (Mb), and wind velocity in meters per second (m/s). Relative humidity (%) was also collected.

### 2.4. Clinical Assessment

The number of painful joints (TJC) and swollen joints (SJC) was extracted from the patients' medical records. Disease activity was calculated by DAS28 using the erythrocyte sedimentation rate (ESR), which was available for the same visit. DAS28ESR is a composite score that includes TJC and SJC of 28 joints, patient global assessment of the disease, and ESR [12]. Remission was defined as DAS28 under 2.6. The intensity of joint pain was evaluated using the visual analogue scale (VAS) at the time of the visit from 0 (no pain) to 10 (maximum pain). The data on the age, gender, presence of associated Sjogren's syndrome, and patients' body mass index (BMI) were also recorded. Sjogren's disease was diagnosed according to the American-European Consensus Group criteria (2002).

### 2.5. Statistical Analysis

The data were analyzed by the IBM® statistical package for social sciences software (version 20). The descriptive analysis of the patients and meteorological parameters during the seasons was made. Correlations using the Pearson test were applied to identify any associations between meteorological parameters and disease variables for each season. Then, we performed a multiple linear regression analysis to study the effect of adjustment of meteorological parameters on disease parameters. The parameters of each season were analyzed separately. The dependent variables were tender joint count, swollen joint count, pain intensity (VAS), and DAS28. Age, BMI, and the presence of Sjogren's syndrome were added to models of regression for all meteorological parameters in a season. Our cutoff value for statistical significance was *p* < 0.05.

## 3. Results

The effect of 6 meteorological parameters on 117 patients during three seasons, which included a total of 346 consultation days (the data for 5 visits were not available), was studied. The average means of meteorological parameters collected for three seasons are shown (Table 1).

The mean age of our patients was 50.45 ± 12.17 years. Women accounted for 86.3%. The majority of our patients were in a remission state of the disease according to their mean DAS28 for the three seasons (Table 2).

Correlation analyses showed a significant association of both tender joint count (*p*=0.027) and pain intensity (*p*=0.01) with humidity in the winter. In the summer, minimal temperature (*p*=0.024) and barometric pressure (*p*=0.04) were associated with pain intensity. However, we observed no significant association between meteorological and RA parameters in the spring (Table 3).

Multiple linear regression analyses indicate that in the summer, temperature and wind speed tend to explain the four RA activity predictors. In the winter, an increase of 1°C in minimal temperature was associated with an increase of 0.504 in TJC. In the summer, an increase of 1°C was associated with a decrease of 0.534 in TJC (Table 4).

The pain intensity (VAS) was associated with Sjogren's syndrome presence only in the summer in multiple linear regression (*p*=0.025; *β* = 0.204 [0.11, 1.64]) whereas BMI and age were not associated with disease activity parameters for all seasons.

## 4. Discussion

These results underscore weather and seasonal effects on RA pain, especially for extreme seasons and extreme temperatures. It was determined that the effect of extreme temperatures in the summer and winter was significant. Our RA patients were more likely to report tender joints with minimal temperature increase and maximal temperature decrease in the winter. The inverse effect was observed in the summer; specifically, patients had more painful joints when the daily minimum temperature decreased and the maximum temperature increased. The effect of temperature on RA pain has been previously reported [13, 14]; however, none of these reports discussed the extreme daily temperature effect for each season. Still, Abasalo et al. in their case-crossover study have used seasonality to explain the worsening of RA patients' symptoms at mean extreme temperatures (<10°C and >20°C) [14]. In addition, temperature was associated with TJC in the longitudinal study by Savage et al. [15]. This seasonal effect may explain the absence of any association between rheumatic pain and meteorological parameters in other studies, where the study time was insufficient to assess seasonal variations [16, 17]. Additionally, the objective parameters of the disease were insensitive to meteorological parameter variation during seasons. However, Pearson correlation is a rigorous test, and the results of correlations can be more significant if outliers are ignored (painless patients). In multiple regression analyses, some associations appeared and others disappeared under the effect of the adjustment. These results show an intriguing interrelation between meteorological parameters and the human body. In Patberg's review, the conflicting results in the literature on temperature and RA pain association are explained by the temperature-humidity couple and its effect on the microclimate near the skin [18]. The humidity of microclimate is affected by water evaporation from sweat glands on the skin surface and generates a local vapor pressure. In a dry climate like ours, the vapor pressure is kept high in the winter owing to the use of clothing and associated warming. However, in the summer, the vapor pressure is lower because less clothing is used. The author stated that the high humidity of the microclimate is related to RA pain. This complex relationship between the outdoor climate and microclimate may explain our findings. In addition, the results can be affected by the particular clothing habits of our patients (i.e., veiled women) and the underuse of air conditioning in our area.

In several previous studies, the effect of weather and seasons on disease activity, specifically DAS28, has been observed [15, 19]. In our study, the effect of weather on DAS in the summer can be explained by the relevant observed effect of meteorological parameters on subjective DAS components (TJC and VAS). In addition, the frequent tissue inflation observed in the summer [20] may potentially increase SJC and explain the observed DAS effect.

In this study, there was also a seasonal effect on another autoimmune disease, i.e., “Sjogren's syndrome.” Little is known about how weather may affect rheumatic pain; the cognitive and psychological effect of weather on pain is a largely discussed hypothesis in the literature [10, 15, 17]. It has been also suggested that some individuals are more sensitive to weather than others [21]. A possible seasonal effect on vitamin D level was advanced; however, no evidence of such association was observed in the studies [22]. Another proposed mechanism is the effect of weather parameters (barometric pressure and temperature) on tissues (e.g., tendons or muscles), which may affect sensitized nerve pain by the induced expansion and contraction [23].

In this study, there were several limitations that are usually encountered in a retrospective design. We did not know whether our patients traveled out of the city during the studied period, and we could not evaluate differences between outdoor and indoor climate. In addition, it was regrettable that other confounding factors (e.g., osteoarthritis or fibromyalgia) could not be studied. Despite these limitations, the retrospective design allowed to avoid the patients' and physicians' awareness bias. In addition, this is the first study from our region, which contains objective and subjective measurements, that evaluates RA disease activity with meteorological parameters during three seasons.

## 5. Conclusion

This study showed the possibility of weather effects on RA patients' pain depending on seasons, especially the effect of extreme temperatures. In addition, the presence of Sjogren's syndrome affected pain intensity perception in our patients in the summer. Our results confirm that previous studies in the literature cannot be generalized and should take into consideration seasonal variation, geographic discrepancies within studies, and other factors (e.g., Sjogren's syndrome), which may be the subject of future studies.

## Figures and Tables

**Table 1:** Average means of meteorological parameters of the three seasons during the study time.

	Winter	Spring	Summer
Temperature^*∗*^ (°C) (minimum/maximum)	10/15	15.9/19.5	24.5/32.7
Precipitation^*∗*^ (mm per day)	1.2	3.14	0.08
Humidity^*∗*^ (%)	58.1	71	49.7
Barometric pressure^*∗*^ (Mb)	1023.9	1016.4	1016.9
Wind speed^*∗*^ (m/s)	4.1	6.1	5.9

^*∗*^All variables are given as means.

**Table 2:** Descriptive data of the population.

	*n* = 117		
Age (years)^¶^	50.45 ± 12.17		
Women^ǂ^	101 (86.3%)		
Disease duration (years)^¶^	10.57 ± 8.49		
Sjogren's syndrome (*n* = 109)^ǂ^	51 (46.8%)		
BMI^¶^	26.63 ± 8.04		

Seasons	Winter *n* = 116	Spring *n* = 116	Summer *n* = 114

TJC^¶^	0.82 ± 1.61^*∗*^	1.04 ± 1.99^*∗*^	0.89 ± 2.35^*∗*^
SJC^¶^	0.48 ± 1.14^*∗*^	0.72 ± 1.38∗	0.57 ± 1.41^*∗*^
VAS^¶^	1.36 ± 1.96^*∗*^	1.57 ± 2.06^*∗*^	1.43 ± 2.13^*∗*^
DAS28ESR^¶^	2.44 ± 0.95	2.60 ± 0.98	2.57 ± 0.95

^¶^Mean ± standard deviation; ^ǂ^number (%). ^*∗*^All variables have a median of 0. BMI: body mass index; TJC: tender joint count; SJC: swollen joint count; VAS: patient's pain assessed by the visual analogue scale.

**Table 3:** Correlation coefficients between meteorological parameters and RA variables in studied seasons.

Season	Meteorological parameter	TJC	SJC	VAS	DAS28
Winter	Min-T°	−0.05 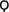	−0.00^ 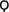 ^	−0.11^ 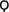 ^	−0.03^ 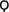 ^
	Max-T°	−0.14^ 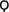 ^	−0.01^ 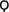 ^	−0.17^ 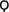 ^	−0.06^ 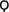 ^
	Humidity	0.20^*∗*^	0.12^ 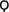 ^	0.24^*∗*^	0.17^ 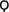 ^
	Precipitation	0.06^ 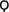 ^	−0.00^ 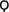 ^	0.11^ 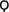 ^	−0.00^ 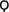 ^
	Wind speed	−0.03^ 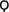 ^	0.09^ 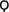 ^	−0.02^ 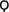 ^	0.07^ 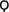 ^
	Barometric pressure	−0.11^ 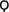 ^	−0.00^ 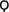 ^	−0.11^ 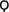 ^	−0.10^ 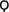 ^

Spring	Min-T°	0.05^ 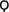 ^	0.09^ 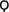 ^	0.08^ 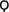 ^	0.08^ 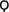 ^
	Max-T°	−0.01^ 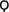 ^	0.00^ 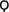 ^	−0.09^ 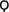 ^	−0.02^ 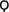 ^
	Humidity	0.15^ 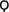 ^	0.15^ 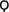 ^	0.07^ 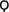 ^	0.12^ 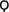 ^
	Precipitation	−0.08^ 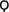 ^	−0.06^ 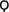 ^	−0.13^ 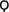 ^	−0.17^ 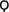 ^
	Wind speed	−0.13^ 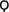 ^	−0.11^ 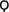 ^	−0.08^ 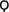 ^	−0.16^ 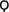 ^
	Barometric pressure	0.00^ 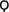 ^	0.03^ 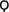 ^	−0.05^ 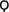 ^	0.03^ 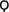 ^

Summer	Min-T°	−0.16^ 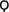 ^	−0.12^ 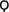 ^	−0.21^*∗*^	−0.05^ 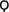 ^
	Max-T°	0.07^ 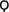 ^	0.06^ 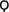 ^	0.14^ 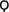 ^	0.08^ 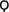 ^
	Humidity	0.09^ 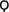 ^	0.06^ 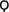 ^	0.12^ 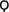 ^	−0.10^ 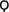 ^
	Precipitations	0.27^*∗*^	0.11^ 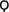 ^	−0.02^ 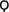 ^	0.08^ 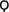 ^
	Wind speed	−0.00^ 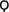 ^	−0.08^ 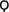 ^	0.13^ 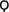 ^	−0.05^ 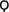 ^
	Barometric pressure	0.07^ 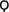 ^	0.04^ 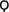 ^	−0.19^*∗*^	0.01^ 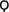 ^

^*∗*^Pearson correlation, *p* < 0.05. ^
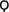
^Pearson correlation, *p* > 0.05. TJC: tender joint count; SJC: swollen joint count; VAS: patient's pain assessed by the visual analogue scale; Min-T°: minimal temperature; Max-T°: maximal temperature.

**Table 4:** Multiple linear regression coefficients.

	TJC		VAS		SJC		DAS28	
	*β*	95% CI	*β*	95% CI	*β*	95% CI	*β*	95% CI
*Winter*								
Minimal T°	0.504^*∗*^	0.01, 0.49	0.161	−0.19, 0.39	−0.006	−0.18, 0.17	0.083	−0.12, 0.18
Maximal T°	−0.664^*∗*^	−0.55, −0.01	−0.203	−0.44, 0.23	0.076	−0.18, 0.22	−0.131	−0.21, 0.13
Humidity	0.122	−0.01, 0.03	0.222	−0.00, 0.05	0.184	−0.01, 0.03	0.174	−0.01, 0.03
Wind speed	−0.085	−0.07, 0.03	−0.132	−0.10, 0.03	0.089	−0.02, 0.05	0.054	−0.03, 0.04
Precipitations	−0.196	−0.37, 0.09	−0.015	−0.3, 0.274	−0.046	−0.19, 0.15	−0.191	−0.23, 0.06
Barometric pressure	−0.101	−0.07, 0.03	−0.059	−0.08, 0.04	0.063	−0.03, 0.05	−0.076	−0.04, 0.02
*R* ^2^	0.09	

*Spring*								
Minimal T°	0.055	−0.12, 0.17	0.236	−0.03, 0.26	0.180	−0.04, 0.16	0.117	−0.04, 0.10
Maximal T°	0.366	−0.18, 0.08	−0.399^*∗∗*^	−0.32, − 0.06	−0.105	−0.12, 0.06	−0.206	−0.11, 0.02
Humidity	0.157	−0.01, 0.06	0.012	−0.04, 0.04	0.248^*∗*^	0.00, 0.05	0.165	−0.01, 0.03
Wind speed	−0.071	−0.06, 0.03	−0.035	−0.05, 0.04	0.025	−0.03, 0.03	−0.061	−0.03, 0.02
Precipitations	−0.114	−0.12, 0.04	−0.209	−0.15, 0.00	−0.055	−0.07, 0.04	−0.212	−0.07, 0.00
Barometric pressure	0.023	−0.10, 0.12	−0.098	−0.16, 0.07	0.158	−0.03, 0.13	0.080	−0.04, 0.07

*Summer*								
Minimal T°	−0.534^*∗∗*^	−0.38, − 0.09	−0.699^*∗∗∗*^	−0.41, − 0.14	−0.593^*∗∗*^	−0.24, − 0.06	−0.631^*∗∗*^	−0.18, −0.04
Maximal T°	0.471^*∗∗∗*^	0.13, 0.40	0.420^*∗∗*^	0.08, 0.34	0.442^*∗∗*^	0.06, 0.23	0.346^*∗*^	0.01, 0.14
Humidity	−0.056	−0.04, 0.03	−0.088	−0.04, 0.02	−0.019	−0.02, 0.02	−0.334^*∗*^	−0.03, −0.003
Wind speed	−0.287^*∗*^	−0.24, − 0.01	−0.328^*∗*^	−0.24, − 0.02	−0.453^*∗∗*^	−0.19, − 0.05	−0.302^*∗*^	−0.10, −0.003
Precipitations	0.293^*∗∗*^	0.94, 4.16	−0.092	−2.19, 0.75	0.074	−0.62, 1.39	0.092	−0.39, 1,05
Barometric pressure	0.141	−0.06, 0.37	−0.213^*∗*^	−0.40, − 0.01	0.042	−0.11, 0.16	−0.058	−0.12, 0.07
*R* ^2^	0.21		0.198		0.15		0.12	

^*∗*^ *p* < 0.05, ^*∗∗*^*p* < 0.01, and ^*∗∗∗*^*p* < 0.001. TJC: tender joint count; VAS: patient's pain assessed by the visual analogue scale; SJC: swollen joint count; T°: temperature.

## Data Availability

The data that support the findings of this study are available from the corresponding author upon reasonable request.
